# Effect of apolipoprotein E polymorphism on cognition and brain in the Cambridge Centre for Ageing and Neuroscience cohort

**DOI:** 10.1177/2398212820961704

**Published:** 2020-10-07

**Authors:** Richard N. Henson, Sana Suri, Ethan Knights, James B. Rowe, Rogier A. Kievit, Donald M. Lyall, Dennis Chan, Else Eising, Simon E. Fisher

**Affiliations:** 1 MRC Cognition & Brain Sciences Unit, University of Cambridge, Cambridge, UK; 2 Department of Psychiatry, University of Cambridge, Cambridge, UK; 3 Department of Psychiatry, Warneford Hospital, University of Oxford, Oxford, UK; 4 Wellcome Centre for Integrative Neuroimaging, University of Oxford, Oxford, UK; 5 Department of Clinical Neurosciences, University of Cambridge, Cambridge, UK; 6 Institute of Health and Wellbeing, University of Glasgow, Glasgow, UK; 7 Institute of Cognitive Neuroscience, University College London, London, UK; 8 Language and Genetics Department, Max Planck Institute for Psycholinguistics, Nijmegen, The Netherlands; 9 Donders Institute for Brain, Cognition and Behaviour, Radboud University, Nijmegen, The Netherlands

**Keywords:** Cognition, apolipoprotein E, lifespan, brain, ageing

## Abstract

Polymorphisms in the apolipoprotein E (APOE) gene have been associated with individual differences in cognition, brain structure and brain function. For example, the ε4 allele has been associated with cognitive and brain impairment in old age and increased risk of dementia, while the ε2 allele has been claimed to be neuroprotective. According to the ‘antagonistic pleiotropy’ hypothesis, these polymorphisms have different effects across the lifespan, with ε4, for example, postulated to confer benefits on cognitive and brain functions earlier in life. In this stage 2 of the Registered Report – https://osf.io/bufc4, we report the results from the cognitive and brain measures in the Cambridge Centre for Ageing and Neuroscience cohort (www.cam-can.org). We investigated the antagonistic pleiotropy hypothesis by testing for allele-by-age interactions in approximately 600 people across the adult lifespan (18–88 years), on six outcome variables related to cognition, brain structure and brain function (namely, fluid intelligence, verbal memory, hippocampal grey-matter volume, mean diffusion within white matter and resting-state connectivity measured by both functional magnetic resonance imaging and magnetoencephalography). We found no evidence to support the antagonistic pleiotropy hypothesis. Indeed, Bayes factors supported the null hypothesis in all cases, except for the (linear) interaction between age and possession of the ε4 allele on fluid intelligence, for which the evidence for faster decline in older ages was ambiguous. Overall, these pre-registered analyses question the antagonistic pleiotropy of APOE polymorphisms, at least in healthy adults.

## Introduction

Apolipoprotein E (APOE) is a protein that plays an important role in lipid metabolism (including cholesterols) and has been implicated in synaptogenesis, repair of injured nerve tissue and the modulation of beta-amyloid plaques and neurofibrillary tangles that characterise Alzheimer’s disease (AD) (for review, see Belloy et al., 2019; Rocchi et al., 2003). The gene coding for APOE is located on chromosome 19 and is polymorphic in the general population. The three most common alleles are ε2, ε3 and ε4, with approximate allele frequencies of 6%, 78% and 15% in healthy Caucasian Europeans (Eisenberg et al., 2010). Possession of the ε4 allele has been associated with poorer cognitive abilities and more rapid longitudinal decline in healthy older people, particularly in episodic memory (e.g. Jack et al., 2015; Jochemsen et al., 2012; Jorm et al., 2007; Lyall et al., 2016; Marioni et al., 2016; Mondadori et al., 2007; Schiepers et al., 2012; Schultz et al., 2008; Shin et al., 2014; see Wisdom et al., 2011 for a meta-analysis). It has also been associated with a three-to-fourfold increase in the risk of late onset AD in a gene dose-dependent manner (Farrer et al., 1997) and with an earlier age at onset by nearly 6 years on average for ε4 carriers (Blacker et al., 1997). Indeed, some have argued that APOE has no influence on cognition in mid- or late-life beyond increasing risk for AD, such that effects found on cognition reflect the decades of a pre-symptomatic period of AD pathology (e.g. Vemuri et al., 2010; see also O’Donoghue et al., 2018).

**Figure 1.**
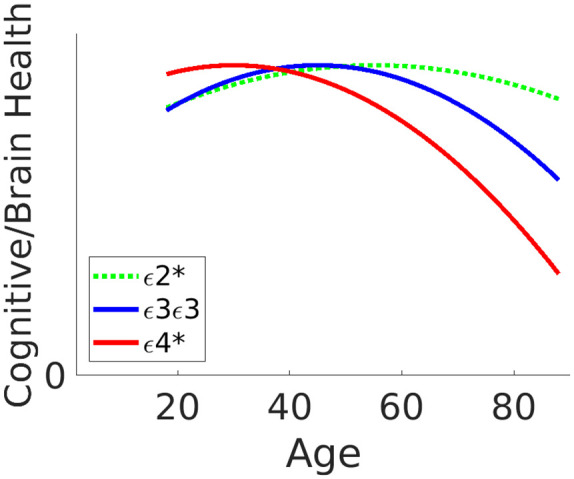
Illustration of potential interactions between APOE status and age on cognitive and/or brain health. According to the ‘antagonistic pleiotropy’ hypothesis, ε4-carriers (red line) experience a benefit in early life but a detriment in later life, relative to non-carriers (ε3ε3 group, blue line). The characteristics of ε2-carriers (green line) are less clear, but generally thought to benefit later in life. Note that every person has two alleles, so ‘ε4-carrier’ (indicated by ‘ε4*’ in legend) means someone with ε4ε4 or ε3ε4 (but excluding ε4ε2 – see text for more details).

There have also been claims that the ε4 allele confers cognitive benefits earlier in life, that is, individuals aged 5–35 years (e.g. Puttonen et al., 2003; Rusted et al., 2013; Yu et al., 2000, though, see Ihle et al., 2012 for a meta-analysis of studies that found no consistent effect of ε4 status on cognitive tests in this age-group). These claims led to the ‘antagonistic pleiotropy’ hypothesis of Han and Bondi (2008), whereby the APOE ε4 allele is hypothesised to be advantageous in early life but disadvantageous in later life (and potentially neutral in middle-age, consistent with the lack of association with cognition in a meta-analysis of middle-aged people by Lancaster et al., 2017). The antagonistic pleiotropy hypothesis is illustrated in Figure 1 (cf. red versus blue lines).

Cognitive benefits in early life (while still fertile), or impairments that only arise later in life, might explain in evolutionary terms why the ε4 allele persists in the population at a relatively high frequency (Fullerton et al., 2000). However, most studies of ε4 have examined young, middle-aged or older samples separately. Moreover, studies of older participants are often biased by the over representation of super-healthy individuals who are motivated to respond to adverts or join volunteer panels, as opposed to population-based recruitments.

To examine the effect of APOE across the adult lifespan, we used the ‘imaging’ sample of Cambridge Centre for Ageing and Neuroscience (CamCAN; www.cam-can.org) to test for a continuous interaction between age and APOE status on a variety of cognitive and brain measures. CamCAN is a population-derived, lifespan cohort of healthy adults uniformly distributed from 18 to 88 years, which allows us to overcome many of the limitations of former studies of the effects of ε4 at different ages.

Another strand of the literature has examined the possible protective effects of the ε2 allele, which has been reported to reduce risk of AD (Farrer et al., 1997). The relationship of these putative protective effects with age is less well studied (partly due to its rarer frequency), but one suggestion is that ε2 is associated with more robust neurodevelopment in early life (relative to ε3), while another is that it is associated with lower levels of neurodegeneration (relative to ε4) in later life (Suri et al., 2013). CamCAN’s lifespan cohort allows novel tests for these interactive or additive effects of the ε2 allele with age (green line in Figure 1).

**Figure 2.**
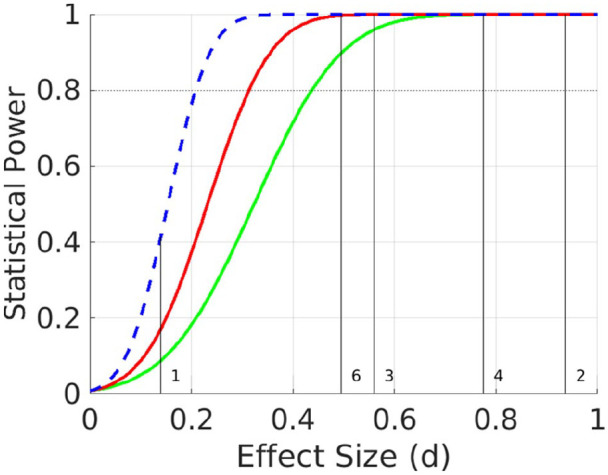
Classical power analyses for (any) polynomial term of GLM for (1) separate, categorical analysis of ε3ε3 versus ε2+ε4− (green line), (2) separate, categorical analysis of ε4+ε2− versus ε3ε3 (red line) and (3) linear, parametric ‘dose’ effect of ε2−/ε4+ load (blue dotted line). The vertical lines and numbers refer to prior effect sizes in literature, as described in text.

The mean effect size of ε4-carrier versus non-carrier status on cognition is typically small when averaging across ages. For example, the meta-analysis of Wisdom et al. (2011) showed a Cohen effect size (d) across studies of d = −0.14 on episodic memory and d = −0.05 on global cognition, averaged across ages from 20 to 90 years (where d = 0.20 is considered ‘small’). This effect size would be difficult to detect with the approximately 600 participants in the CamCAN imaging sample who have valid cognitive, neuroimaging and genetic data, including APOE status (see Figure 2); a sample that is considerably smaller than several recent large studies (e.g. Lyall et al., 2019a; Marioni et al., 2016; Shin et al., 2014). However, if the antagonistic pleiotropy hypothesis is correct, then the effect size depends on age, such that the effect size for the interaction between APOE and age could be bigger than the effect size averaging over age (see Figure 1 and section ‘Methods’). Consistent with this view, the meta-analysis of Wisdom et al. (2011) also found a significant linear effect of age, with the detrimental effect of ε4 increasing with age. Similarly, more recent studies (e.g. Lyall et al., 2019b; Marioni et al., 2016; Shin et al., 2014) have generally reported stronger detrimental effects of ε4 in older than younger groups. Moreover, in a large (genome-wide) study of multiple cohorts, Davies et al. (2015) found that rs10119 – a single-nucleotide polymorphism (SNP) neighbouring the APOE locus – showed a negative correlation of R = −0.424 between the average cohort age (from 55 to 80 years) and the effect size for this SNP on general cognitive ability. This corresponds to an interaction effect size of d = −0.936 (Figure 2). Furthermore, the power to detect such an interaction between age and APOE status is likely to benefit from the wider age range in CamCAN than is typically available in the cohorts that have been tested so far (such as the UK Biobank), which have tended to focus on middle and older ages.

In addition to cognition, the APOE variants have been associated with differences in brain structure and function, as measured with magnetic resonance imaging (MRI) or magnetoencephalography (MEG). Indeed, it is possible that such allele-specific effects might be seen earlier in the lifespan than they can be seen in cognition (Siebner et al., 2009). This could be because neuroimaging measures are more sensitive than behavioural measures at detecting subclinical abnormalities. Another possibility for the lower sensitivity of cognitive measures (versus brain measures) is that young ε4-carriers show compensatory changes (e.g. increased functional activity or connectivity) that maintain cognitive performance comparable to non-carriers; however, at some point in older age, these compensatory mechanisms fail and a deleterious effect on cognition is unmasked. A related idea is that the increased activity in younger ε4-carriers ‘may cause neurophysiological changes that lead to earlier age-related decline in brain function’ (Buckner et al., 2008; Filippini et al., 2009a). Both of these hypotheses predict an interaction between APOE variant and age. Indeed, if one were to find a greater interaction between APOE and age on brain measures than on cognitive measures, this would support the idea that brain function compensates for the detrimental effects of APOE on brain structure, compensating in youth but no longer being able to compensate in old age.

Many neuroimaging studies of APOE variants have focused on volumetric differences in structural (e.g. T1-weighted) MRI images, particularly atrophy of grey matter in regions of the medial temporal lobe, such as the hippocampus, which occurs early in AD and is associated with memory problems. Unfortunately, the results have been mixed, with most studies not finding a mean effect of APOE, ε4 or ε2, particularly in hippocampus (e.g. Bunce et al., 2012; Filippini et al., 2009b; Habes et al., 2016; Jack et al., 2015; Lyall et al., 2019b; Machulda et al., 2011; Westlye et al., 2011; Taylor et al., 2015; see Table 1 in Lyall et al., 2013 and Fouquet et al., 2014 for review). Nonetheless, despite the converging evidence against any effect of APOE, we tested main effects on hippocampal volume and the interaction with age because of their historical and theoretical relevance.

**Table 1.** Number of outliers and valid data points for each phenotypic variable for each of the three genetic groups: ε2+ε4−, ε3ε3 and ε4+ε2−.

Count	N initial	N outliers (excluded)				N final			
Phenotypic variable		ε2+ε4−	ε3ε3	ε4+ε2−	Total	ε2+ε4−	ε3ε3	ε4+ε2−	Total
Fluid intelligence	560	0	2	1	3	74	331	152	557
Episodic memory	592	0	1	1	2	78	355	157	590
Hippocampal volume	546	1	4	7	12	70	322	142	534
White-matter MD	525	7	26	12	45	63	286	131	480
rsfMRI in DMN	548	0	6	3	9	71	322	146	539
rsMEG PCA	514	2	7	7	16	67	295	136	498

MD: mean diffusivity; rsfMRI: resting-state functional magnetic resonance imaging; DMN: default mode network; rsMEG: resting-state magnetoencephalography; PCA: principal component analysis.

Other MRI studies have examined effects of APOE on white-matter integrity, for example, the number of white-matter hyper-intensities (WMHI) in MRI images (e.g. Lyall et al., 2019b). While CamCAN does not have MRI data suitable for estimating WMHI in the full sample, it does possess diffusion-weighted images (DWIs) that can be used to estimate diffusion tensor imaging (DTI) metrics. Given APOE’s established role in cholesterol transport, it is conceivable that different alleles may modulate age-related myelin degradation, which would affect such DTI metrics. Two studies (Heise et al., 2011; Westlye et al., 2012) with samples of 73 and 203 adults, respectively, found similar effects of ε4- and ε2-carriers (relative to ε3ε3) on various DTI metrics, including increases in mean diffusivity (MD) and decreases in fractional anisotropy (FA), where both of these changes are reflective of poorer WM health. Similar MD increases and FA decreases have been reported in AD (Stebbins and Murphy, 2009). However, the parallel rather than opposite effect of ε4 and ε2, coupled with the fact that neither study found any interactions with age, led both groups to suggest that the APOE variants reflected neurodevelopmental differences, rather than any changes related to AD. A study of N ~ 650 older individuals (aged ~73 years) found reduced FA in ε4-carriers (but not ε2-carriers) in two of 15 major white-matter tracts, but not in the first principal component across tracts (Lyall et al., 2014). A more recent and much larger study from the UK Biobank (Lyall et al., 2019b) found neither effect of ε4 nor interaction with age, on the first principal component of MD or FA across 22 WM tracts. However, as the authors noted, the restricted age range of mostly 50–70 years may have limited their ability to detect age effects. CamCAN’s larger range of ages make it better suited to investigate the effects of ε4 and ε2 and their interactions with age.

Several studies have assessed functional connectivity between brain regions, acquiring, for example, blood oxygenation level dependent (BOLD)-weighted MRI while people rest. There has been a focus on the default mode network (DMN), which includes hippocampus as well as medial parietal and medial frontal regions. Using independent component analysis (ICA), for example, Westlye et al. (2011) reported increased expression of DMN connectivity for N = 33 ε4-carriers versus N = 62 non-carriers (aged 50–80 years). Based on the similarity of the DMN and the regions disrupted in AD, it has been suggested that enhanced metabolism in the DMN may provide regional conditions that are conducive to amyloid deposition (Buckner et al., 2008). However, Trachtenberg et al. (2012) found similar effects of ε4 and ε2 on functional connectivity in a young group and argued that the effects of APOE do not relate only to AD risk, but rather to the putative role of APOE in neurodevelopment (see also Deary et al., 2002; O’Donoghue et al., 2018). Moreover, other results are divergent, with both increased and decreased functional connectivity reported for APOE variants (e.g. Damoiseaux et al., 2012; Filippini et al., 2009a; Fleisher et al., 2009; Machulda et al., 2011; Pietzuch et al., 2019; Sheline et al., 2010 for review). Indeed, Pietzuch et al. (2019) suggested that this divergence ‘may relate to the age of the sample groups’ (see also Damoiseaux et al., 2012 for potential moderating effects of age and sex). Indeed, in a cross-sectional study, Shu et al. (2016) reported that, while both ε2- and ε4-carriers had decreased DMN connectivity compared to ε3 homozygotes, they showed opposite effects of age.

Interpretation of changes in functional connectivity measured by functional MRI (fMRI) is non-trivial, since the measures can be affected by vascular as well as neural factors (Geerligs et al., 2017; Tsvetanov et al., 2016). MEG provides a more direct measure of neural activity, albeit with worse spatial resolution than fMRI. To our knowledge, Koelewijn et al. (2019) is the only MEG study to examine resting-state functional connectivity in APOE variants with an appreciable number of N = 159 healthy adults (N = 159; 51 ε4-carriers versus 108 non-carriers aged 18–65 years, though mostly young; see Cuesta et al., 2015, for a smaller study). These authors reported that they could classify ε4 status with an accuracy of 63.5%, based on the strongest connections between brain regions. Though the CamCAN data were recorded with eyes closed rather than open and on a different MEG system, we replicated their analysis pipelines, as closely as possible, for the CamCAN data.

In summary, we tested the antagonistic pleiotropy hypothesis, with regard to both ε4- and ε2-carriers, on a number of cognitive and brain measures that are available in the CamCAN cohort; specifically, those measures that have been linked to APOE status in previous studies, empirically and/or theoretically. We used a quadratic expansion of age (see section ‘Methods’) to test for interactions between APOE status and age. In the case of significant non-linear effects (e.g. inverted U-shaped fits as in Figure 1), we planned to estimate the age of the vertex (peak scores) for each allele group. Since it was possible that we would identify no significant quadratic component (if, for example, the beneficial effects of ε4 or ε2 are only seen below the age of 18 years, that is, during early development), such that any interaction between age and APOE status could reflect just an accelerated decline in old age, we also compared linear slopes.

## Methods

### Data selection

None of the CamCAN participants had a diagnosis of dementia or mild-cognitive impairment at recruitment; all reported themselves to be in good cognitive health, and all scored above conventional cut-offs for dementia on the mini-mental state examination (MMSE) and Addenbrooke’s cognitive examination – revised (ACE-R) screening tests (see Shafto et al., 2014). This study was conducted in compliance with the Helsinki Declaration and was approved by the local ethics committee, Cambridgeshire 2 Research Ethics Committee (reference: 10/H0308/50). The cognitive, MRI and MEG data from CamCAN are available on request from https://camcan-archive.mrc-cbu.cam.ac.uk/dataaccess/. The cognitive data were already scored (available on above website) and the MRI data had already been preprocessed (see Taylor et al., 2017).

DNA was prepared from saliva samples, which underwent genome-wide genotyping using the Illumina Infinium ‘OmniExpressExome’ SNP-chip. This chip covers >960,000 SNP markers spread through the genome, capturing a large proportion of common variation. The common SNPs rs7412 and rs429358 were used to determine APOE ε2, ε3 and ε4 allelic status of the participants. Full raw genotype data were first filtered in GenomeStudio according to standard procedures (Guo et al., 2014). Additional quality control checks were performed in PLINK (removal of SNPs for which Hardy Weinberg p < 1 × 10^−6^, missingness > 0.05, minor allele frequency < 0.05; removal of individuals with total SNP missingness > 0.05 or where multidimensional scaling indicated non-European origin). After quality control, the data set included 675,373 directly genotyped SNPs. Genotype data were imputed using the Haplotype Reference Consortium version 1.1 panel in the Michigan Imputation Server (https://imputationserver.sph.umich.edu). Genotypes of rs7412 were extracted from the raw genotype data, while genotypes for rs429358 were derived by imputation. Prior studies have shown that this imputation procedure has high accuracy for determining APOE allelic status (Radmanesh et al., 2014). The phenotypic and genotypic data were held in separate laboratories (MRC CBU in Cambridge and Max Planck Institute for Psycholinguistics in Nijmegen, respectively), and the link between them only de-blinded when the Stage 1 version of this study was accepted and restricted to APOE allelic status.

### Choice of dependent variables and models

In order to minimise the number of statistical tests, we restricted ourselves to six dependent variables – two cognitive and four brain measures – which we deemed to have the strongest prior evidence for associations with APOE allelic status (e.g. episodic memory) or are most theoretically relevant (e.g. hippocampal volume). These measures also have prior effect sizes published in the literature with which to estimate power (see section ‘Power calculation’ below).

For the cognitive data, we focused on two abilities that have shown the most consistent associations with APOE status in the literature: (1) fluid intelligence and (2) episodic memory (Davies et al., 2015; O’Donoghue et al., 2018; Wisdom et al., 2011). For the former, we used the Cattell test (first principal component across four sub-tests) and for the latter we used the WAIS logical memory test of verbal memory (first principal component across immediate recall, delayed recall and delayed recognition) – see Shafto et al. (2014) for more details.

For grey matter, we focused on (3) hippocampal volume, as extracted following application of FreeSurfer 6.0 (https://surfer.nmr.mgh.harvard.edu/fswiki/DownloadAndInstall) to 1 mm-isotropic T1-weight magnetization prepared rapid gradient echo (MPRAGE) scans (see https://camcan-archive.mrc-cbu.cam.ac.uk/dataaccess/pdfs/CAMCAN700_MR_params.pdf for further details of MRI scans). To correct for inter-individual differences in head size, hippocampal volumes were adjusted for total intracranial volume (TIV).

For white matter, we used (4) the participant loadings of the first principal component of MD values across major white-matter tracts defined by the John Hopkins Atlas (averaged across hemisphere, as in de Mooij et al., 2018), derived from 2-mm-isotropic DWI data preprocessed according to Taylor et al. (2017).

For resting-state fMRI (rsfMRI) connectivity, we focused on (5) mean connectivity within the DMN, following the optimised pre-processing pipeline described in Geerligs et al. (2017).

Finally, for the resting-state MEG (rsMEG) connectivity, we followed the procedure of Koelewijn et al. (2019) and examined (6) the participant loadings of the first principal component of connection strengths in source space. For further details of the preprocessing of the imaging measures and minor deviations from the Stage 1 report, see Supplemental Table 1.

### Statistical models

For all six dependent variables, we modelled the effects of age by a second-order polynomial expansion, implemented in a general linear model (GLM). A standard quadratic expansion of age is given by

$$y=\beta_{0}+\beta_{1}x+\beta_{2}x^{2}$$

wherey is the dependent variable (e.g. cognitive score), x is (mean-corrected) age and $\beta_{0-2}$ are the polynomial parameters to be estimated. Note that there is an equivalent parabolic form

$$y={\tilde{\beta }}_{0}+{\tilde{\beta }}_{1}{\left(x-{\tilde{\beta }}_{2}\right)}^{2}$$

in which ${\tilde{\beta }}_{2}$ is the vertex (age of maximal performance as shown in Figure 1), which can be estimated as

$${\tilde{\beta }}_{2}=-\beta_{1}/2\beta_{2}$$

All regressors of interest in the GLM were Z-scored. The GLMs were fit in the MATLAB function ‘apoe_getdata.m’ and equivalently in the R script ‘apoe_lm_brms.R’, available on https://osf.io/ehs9n/. Outliers on any of the six phenotypic variables were defined by residuals that were 1.5 times the interquartile range, after adjusting for polynomial effects of age.

In the ‘categorical’ GLMs, the effects of age were modelled separately for three sub-groups (Table 1): ε2-carriers without ε4, or henceforth the ‘ε2+ε4−’ group (i.e. ε2ε2, ε2ε3); ε4-carriers without ε2, or henceforth the ‘ε4+ε2−’ group (i.e. ε3ε4, ε4ε4) and the ‘ε3ε3’ reference group who do not carry either ε2 or ε4 (we ignored any ε2ε4 cases, which were less than 3% of our sample, as is common in the field).

Two comparisons of pairs of groups were planned: (1) ε3ε3 versus ε2+ε4− and (2) ε4+ε2− versus ε3ε3. The mean and linear age terms were tested separately as one-tailed t-contrasts, where the tail of the test depended on the direction of the expected effect on the specific phenotypic variable (e.g. a detrimental effect of ε4 would produce lower Cattell scores of fluid intelligence, but higher values of MD scores of white-matter integrity). For scores like Cattell, where larger values mean better performance, one would expect a negative difference for both planned comparisons, that is, the ε3ε3 group minus the ε2+ε4− group (since ε2 is hypothesised to be beneficial) and the ε4+ε2− group minus the ε3ε3 group (since ε4 is hypothesised to be detrimental). This direction applies to both the mean and the linear slope of the polynomial effect of age (e.g. the slope of the age effect should be more negative for the ε3ε3 group than the ε2+ε4− group and for the ε2+ε4− group than the ε3ε3 group). The quadratic term was predicted, on the basis of the antagonistic pleiotropy hypothesis, to be negative for all groups (i.e. an inverted U-shape), but can be combined with the mean and linear terms to estimate the vertex (inflexion point) of a parabolic fit (Figure 1), as detailed above, which was predicted to be earlier for ε4-carriers than non-carriers. The above categorical analyses allowed the effects of ε4 and ε2 to differ qualitatively.

A second ‘parametric’, or gene-dose, GLM modelled a linear effect of a decreasing number of ε2 alleles and increasing numbers of ε4 alleles, which is potentially a more sensitive model if the two alleles have the same quantitative (and additive) effects. The latter is consistent with evidence for a load effect for ε4 at least (e.g. Wisdom et al., 2011), including the cumulative increased risk for AD (Farrer et al., 1997).

### Power calculation

We simulated statistical power for various effect sizes and N = 608 participants. The number of 608 was an estimate of the number of CamCAN individuals who have valid cognitive and genetic data; an estimate made before the genetic and phenotypic data were combined (see section ‘Results’ for final numbers for each phenotypic variable). To simulate random genetic sampling, multiple random draws were simulated from the expected population frequencies of each allele combination. The ‘apoe_power_simulations.m’ MATLAB script for these analyses is provided in the website: https://osf.io/ehs9n/. Using a Bonferroni-corrected alpha value of 0.05/6 (given the six outcome variables above – no correction was made for the three contrasts of APOE groups, because these contrasts share subsets of the same data, nor for the number of polynomial effects, since the main interest was in non-zero terms that were relevant to the antagonistic pleiotropy hypothesis), the cohort provides >80% power for effect sizes between 0.20 and 0.44 (i.e. small to medium effects), depending on the comparison and model (Figure 2; note that although we use ‘power’ to refer to a single study, strictly it refers to the expected outcome over a series of studies). The following effect sizes from the literature are shown in Figure 2:

1. The effect size of d = −0.14 of ε4 on episodic memory when averaging across all adult ages (Wisdom et al., 2011), for which our power is only 17% for ε4-carriers versus non-carriers or 41% if there is a parametric effect of ε2−/ε4+ load.
2. More important for the present hypothesis about age-dependence of ε4 effects, the recent meta-analysis of Davies et al., 2015 found an effect size of d = −0.936 for the linear effect of age for the rs10119 region associated with APOE on general cognitive ability, for which the present power is close to 100% in all cases.
3. For hippocampal volume, Taylor et al. (2015) reported an effect size for ε4 of d = −0.56, for which this study has a power of close to 100% (and 95% for ε2 if the effect is comparable in size).
4. For MD from DTI, Westlye et al. (2012) reported an MD effect size for ε4 of d = +0.77, for which the present power is close to 100% in all cases.
5. For rsfMRI, Westlye et al. (2011) reported an effect size for ε4 of d = +1.23 in right hippocampus/amygdala (part of DMN), for which we have ample power (off the scale in Figure 2). Note that, however, the effect sizes for this functional connectivity effect and for the DTI effect in the above Westlye et al.’s paper may be biased upwards because both were selected after thresholding voxels that showed a basic effect of APOE status.
6. For rsMEG, Koelewijn et al. (2019) reported an area-under-curve of the receiver-operating characteristic (ROC) of 63.5% for classification of ε4 status. This corresponds to a Cohen’s d of +0.49 (Salgado, 2018), for which this study has a power of close to 100%.

### Bayes factors

For the same tests on the six dependent variables, we also calculated the Bayes factors (BFs) for the null versus alternative hypothesis for the APOE-by-age interaction terms. For this, we used the Savage–Dickey ratio, after logspline interpolation of the posterior sampling distribution, generated from 100,000 Markov chain Monte Carlo (MCMC) iterations of the ‘brm’ function from the ‘brms’ R package (Bürkner, 2017, 2018) using the same linear model as for the above classical statistics. This is provided in the R script ‘apoe_lm_brms.R’ on https://osf.io/ehs9n/. We imposed unit normal priors on all of the (Z-scored) polynomial terms for the age-by-APOE status interactions. Note that this is a deviation from Stage 1 of this study, which stated that we would use Jeffreys-Zellner-Siow (JZS) priors. This is because the sampling of a Cauchy prior on the mean (with scale 1/√2), as required by the JZS approach, produced unstable posterior distributions. Nonetheless, simulations show that the BFs are very similar for both priors (see https://jaquent.github.io/post/comparing-different-methods-to-calculate-bayes-factors-for-a-simple-model/).

### Covariates of no interest

Covariates of no interest included: (1) sex (which, though balanced in CamCAN sample, has been shown to modulate APOE effects in some studies, e.g. Damoiseaux et al., 2012): (2) education level (since this tends to increase with year of birth in cross-sectional data and is known to correlate with cognitive measures later in life), (3) an estimate of socio-economic status (SES) and (4) a summary measure of cardiovascular health (first principal component of CamCAN’s measures of blood pressure and electrocardiogram (ECG); see Tsvetanov et al., 2015), since this has also been shown to be affected by APOE (e.g. Lyall et al., 2016; Oberlin et al., 2015). Separate models were fit with versus without covariates because education, cardiovascular health and age covary positively and therefore cannot be disentangled with cross-sectional data. Note that, while the Stage 1 report stated that genetic ancestry would also be a covariate, because it became clear after molecular genetic analysis that the majority of the sample had European white ancestry, we excluded the small number of individuals with a different ancestry from the analyses (see start of section ‘Results’), so it was no longer necessary to include ancestry as a covariate.

SES was estimated by total family income, ranked into five levels (<£18k, <£31k, <£52k, <£100k and >£100k per annum) and had 27 missing values. Education was also ranked into five levels (no qualifications at age 16, practical qualifications at age 16, academic qualifications at age 16, qualifications at age 18 and university degree or higher), with 47 missing values. Cardiovascular health was the first principal component of mean heart rate and heart rate variability (after low and high-pass filtering of the ECG recorded during the MEG session), as well as systolic and diastolic blood pressure, after excluding 169 missing values, respectively. All missing values were replaced by the predictions of a quadratic fit of age to remaining values, and the results Z-scored for each variable.

## Results

**Figure 3.**
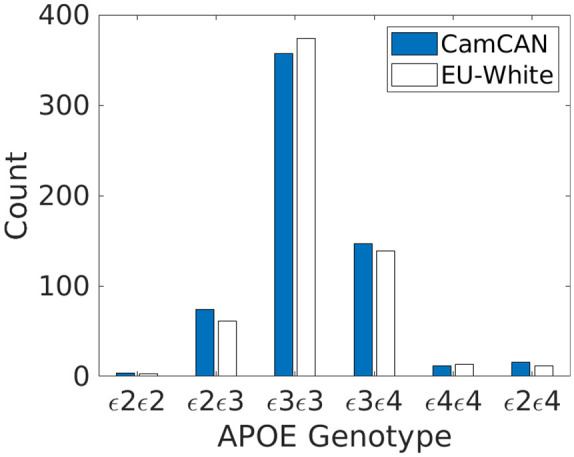
Number of CamCAN participants (out of 610) with each APOE genotype (filled bars) and number expected from White Europeans (empty bars).

There were a total of 651 genetic samples with matching phenotypic IDs, of which three were excluded for low quality and three were excluded because they were related to others in the sample. Of the remaining 645, principal component analyses of genome-wide SNP data showed that only 35 had non-European ancestry, which we also excluded so as to optimise the homogeneity of the sample, leaving a total sample of 610 participants. The frequency of APOE alleles closely matched that expected from the frequency within healthy Europeans (Eisenberg et al., 2010), as seen in Figure 3. There were 78 in the ε2+ε4− group, 159 in the ε4+ε2− group and 357 in the ε3ε3 group (plus 16 ε2ε4 carriers, who were not analysed further, as explained in section ‘Methods’).

The numbers of participants with data for each phenotypic variable are shown in the leftmost column of Table 1. From these, outliers that were 1.5 times the interquartile range, after adjusting for linear and quadratic effects of age, were removed, producing the final numbers in the rightmost column of Table 1. The white-matter MD measure contained the highest number of outliers, most likely because diffusion-weighted MRI data are well-known to be sensitive to noise, such as that caused by head motion.

**Figure 4.**
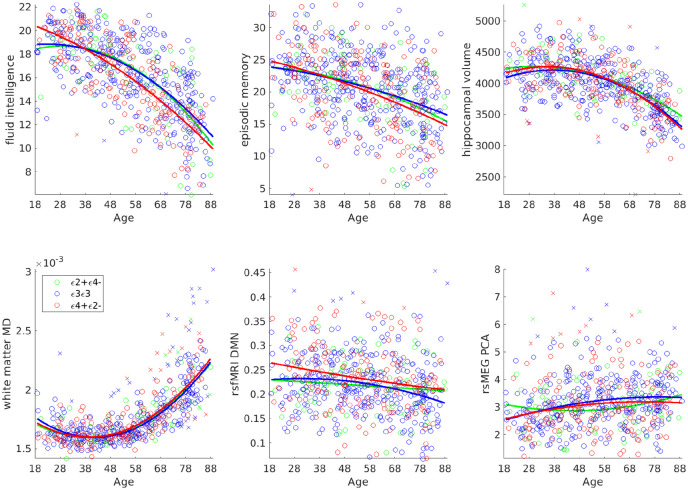
Scatter plots for each phenotypic variable against age, grouped by APOE status (colour). Outliers are indicated by crosses (but were excluded from analyses). The solid lines show second-order polynomial fits of age on remaining points for each APOE group.

Scatter plots of the six phenotypic variables against age are shown in Figure 4. Additional information regarding definition of these variables is provided in Supplemental Table 1. All six variables showed significant effects of age (see Supplemental Table 2). The fits come from a second-order polynomial expansion of age to each APOE group. There was little apparent evidence for antagonistic pleiotropy (i.e. different effects of age for each group), though the Cattell test of fluid intelligence showed some suggestion of ε4-carriers performing worse in later life and better in early life (i.e. for red line relative to blue and green lines in top left panel of Figure 4). Although the quadratic component was significant for three variables, it was never large relative to the linear component, and more importantly, it never differed significantly between APOE groups (see the next section), meaning that there was little value in comparing the peaks across APOE groups (i.e. age of maximum or minimum value).

### Planned comparisons using classical (frequentist) statistics

Results of the critical interactions between APOE and age are shown in Table 2. These come from the same linear model as shown in Figure 4 (without any covariates). In the three groups of columns, the first two groups refer to the two planned categorical contrasts across APOE groups of (1) ε3ε3 versus ε2+ε4−, (2) ε4+ε2− versus ε3ε3; the third group of columns refers to (3) the parametric contrast of ε2−/ε4+ dose, with a linear increase across ε2ε2, ε2ε3/ε3ε2, ε3ε3, ε3ε4/ε4ε3 and ε4ε4. The sign of these three contrasts matches the sign of the expected, prior effect size for ε4-carriers versus non-carriers reported in section ‘Power calculation’ (i.e. the direction of effect was predicted to be negative for first three phenotypic variables and positive for last three phenotypic variables). Within each group, the parameter estimates for the interactions between APOE status and zeroth-, first- and second-order polynomial effects of age are shown (where the zeroth-order effect is equivalent to the main effect of APOE status).

**Table 2.** GLM results for each phenotypic variable (row).

Contrast	ε3ε3 group versus ε2+ε4− group				ε4+ε2− group versus ε3ε3 group				Parametric (dose) effect of ε2−ε4+			
Poly. order	Fit	0	1	2	Fit	0	1	2	Fit	0	1	2
Fluid intelligence	**R**^2^ **=** **0.455** **df** **=** **399**	0.017 (0.122) T = 0.139	0.077 (0.158) T = 0.489	0.072 (0.158) T = 0.458	**R**^2^ **=** **0.500** **df** **=** **477**	−0.133 (0.107) T = 1.246	−**0.228** **(0.115)** **T** **=** **1.982**	0.151 (0.115) T = 1.310	**R**^2^ **=** **0.490** **df** **=** **551**	−0.192 (0.102) T = 1.881	**−0.235** **(0.102)** **T** **=** **2.313**	0.153 (0.102) T = 1.507
Episodic memory	**R**^2^ **=** **0.136** **df** **=** **427**	0.025 (0.244) T = 0.102	0.134 (0.319) T = 0.423	0.072 (0.319) T = 0.225	**R**^2^ **=** **0.145** **df** **=** **506**	−0.102 (0.230) T = 0.443	−0.251 (0.249) T = 1.006	0.023 (0.249) T = 0.926	**R**^2^ **=** **0.145** **df** **=** **584**	−0.138 (0.213) T = 0.648	−0.223 (0.213) T = 1.046	0.026 (0.213) T = 0.121
Hippocampal volume	**R**^2^ **=** **0.406** **df** **=** **386**	−22.12 (14.81) T = 1.494	−2.378 (19.40) T = 0.123	−19.86 (19.40) T = 1.023	**R**^2^ **=** **0.414** **df** **=** **458**	**27.78** **(13.68)** **T** **=** **2.031**	−3.697 (14.85) T = 0.249	2.579 (14.85) T = 0.174	**R**^2^ **=** **0.411** **df** **=** **528**	−7.161 (12.69) T = 0.564	−11.89 (12.70) T = 0.936	−9.45 (12.71) T = 0.743
MD of WM tracts	**R**^2^ **=** **0.710** **df** **=** **343**	8.54e-6 (5.56e-6) T = 1.536	4.34e-6 (7.26e-6) T = 0.597	7.42e-6 (7.26e-6) T = 1.023	**R**^2^ **=** **0.701** **df** **=** **411**	−9.97e-6 (5.16e-6) T = 1.93	−5.25e-6 (5.56e-6) T = 0.945	−4.37e-6 (5.56e-6) T = 0.786	**R**^2^ **=** **0.698** **df** **=** **474**	3.90e-6 (4.82e-6) T = 0.809	4.74e-6 (4.84e-6) T = 0.981	1.43e-6 (4.81e-6) T = 0.298
DMN rsfMRI connectivity	**R**^2^ **=** **0.042** **df** **=** **387**	1.28e-4 (2.84e-3) T = 0.045	−3.82e-3 (3.70e-3) T = 1.032	−2.35e-4 (3.70e-3) T = 0.634	**R**^2^ **=** **0.060** **df** **=** **462**	**7.66e-3** **(2.76e-3)** **T** **=** **2.774**	−1.38e-4 (2.98e-3) T = 0.046	2.83e-3 (2.98e-3) T = 0.951	**R**^2^ **=** **0.046** **df** **=** **533**	4.58e-3 (2.56e-3) T = 1.77	−1.72e-3 (2.56e-3) T = 0.671	−2.78e-4 (2.56e-3) T = 0.914
rsMEG connectivity	**R**^2^ **=** **0.051** **df** **=** **356**	0.059 (0.044) T = 1.362	0.047 (0.056) T = 0.841	−0.083 (0.056) T = 1.486	**R**^2^ **=** **0.042** **df** **=** **425**	−0.046 (0.042) T = 1.098	−0.033 (0.044) T = 0.721	0.005 (0.044) T = 0.116	**R**^2^ **=** **0.034** **df** **=** **492**	0.006 (0.039) T = 0.150	−0.007 (0.039) T = 0.169	−0.031 (0.039) T = 0.793

MD: mean diffusivity; WM: white matter; DMN: default mode network; rsfMRI: resting state functional magnetic resonance imaging; rsMEG: resting state magnetoencephalography; Poly.: polynomial; df: degrees of freedom; R^2^: adjusted R-squared of full model.

The three groups of columns refer to the planned contrasts across APOE groups. Within each group, the first column gives the overall model fit, and the next three columns give the parameter estimates, their associated standard error (in brackets) and unsigned T-statistic for the interaction between the APOE contrast and the three polynomial expansions of age: zeroth (constant), first (linear) and second (quadratic), where zeroth-order term is equivalent to main effect of APOE contrast (see Supplemental Table 2 for parameters for main effects of Age). Effects with p < 0.05 are shown in **bold**, but note that only one survived the pre-specified Bonferroni correction for six multiple, one-tailed comparisons (for which|T| > 2.40 for the minimum number of df’s here; see text), where direction of effect was predicted to be negative for first three phenotypic variables and positive for last three phenotypic variables (see text). These results are without covariates; see Supplemental Table 3 for corresponding results with covariates.

The models fit the data well for the first four phenotypes (explaining 14%–70% of the variance). The fit was not so good for fMRI and MEG (explaining only approximately 5% of the variance, though still significant), which we attribute to these data being noisier. Only one of the three polynomial effects for any of the three contrasts for any of the six phenotypic variables survived our pre-specified, one-tailed alpha value of 0.05/6. This was the zeroth-order effect on resting-state fMRI connectivity in the default mode network, where connectivity was higher on average for the ε4+ε2− group than ε3ε3 group (cf. red and blue lines in middle bottom panel of Figure 4). This is consistent with Westlye et al. (2011), but because this main effect of the ε4 allele did not interact with either of the linear or quadratic age terms, it provides no support for the antagonistic pleiotropy hypothesis.

The only phenotypic variable to show any suggestion of the predicted interaction between APOE status and age was the Cattell measure of fluid intelligence (surviving p < 0.05 but not correction for multiple comparisons), for which the slope was more negative for the ε4+ε2− group than ε3ε3 group (cf. red and blue lines in top left panel of Figure 4), and was negatively related to increasing ε2−/ε4+ dose in the parametric model.

The only other effect with p < 0.05 (uncorrected) was the zeroth-order effect for the ε4+ε2− group on hippocampal volume, which tended to be larger on average than in the ε3ε3 group. However, we suspect that this is a false positive since it was in the opposite direction to our predictions, and so we do not consider it further.

### Adjusting for covariates

The above linear model was repeated with five additional covariates: male/female sex, education level, SES and cardiovascular health. The inclusion of these covariates did not reveal any new significant effects of APOE (Supplemental Table 3). The greater DMN fMRI connectivity for the ε4+ε2− group relative to the ε3ε3 group continued to survive correction, and the parametric effect of dose on the linear age slope for fluid intelligence continued to survive p < 0.05 uncorrected. The linear interaction between age and ε4+ε2− group and ε3ε3 group on fluid intelligence no longer survived p < 0.05 after adjusting for covariates.

In short, there was no evidence for the antagonistic pleiotropy hypothesis using classical null-hypothesis testing. Since this could reflect false negatives, given our relatively small sample for genetic effects, we calculated BFs for the null versus alternate hypotheses.

### BFs

Since the dominant effect of age on phenotypic variables was linear (see Supplemental Table 1), and linear effects were reported in the prior literature (see section ‘Power calculation’), we only report here the BFs for the first-order polynomial term.

The three columns in Table 3 show, for each APOE analysis: (1) BFs for the linear interaction between Age and APOE status being zero, given a prior expectation of zero ( ‘Lin = 0’), (2) BFs for the linear interaction between age and APOE status being greater or less than zero ( ‘Lin > 0’ or ‘Lin < 0’), given a prior of greater or less than zero, where the greater/lesser direction depends on the analysis (i.e. ε2 being protective would predict a more positive (less negative) slope than the reference group, and ε4 being a risk factor would predict a more negative slope than the reference group) and (3) BFs for the linear interaction between age and APOE status being zero given the prior expectation of an effect size (P) equal to that from the literature that was used to power this study ( ‘Lin = P’), as listed in section ‘Power calculation’.

**Table 3.** Bayes factors (BFs) for various hypotheses about the first-order (linear) effect of age being zero (BF01) for each contrast and each phenotypic variable, given various means for a unit normal prior.

Contrast	ε3ε3 group versus ε2+ε4− group			ε4+ε2− group versus ε3ε3 group			Parametric (dose) effect of ε2−/ε4+		
BF01	Lin = 0	Lin < 0	Lin = P	Lin = 0	Lin < 0	Lin = P	Lin = 0	Lin < 0	Lin = P
Fluid intelligence	18.63	29.79	29.42	4.08	2.09	5.99	2.30	1.16	3.31
Episodic memory	15.69	23.30	15.82	13.56	8.05	13.72	13.56	8.05	13.72
Hippocampal volume	19.40	17.71	22.65	25.11	20.95	29.20	19.43	11.79	22.15
BF01	Lin = 0	Lin > 0	Lin = P	Lin = 0	Lin > 0	Lin = P	Lin = 0	Lin > 0	Lin = P
MD of WM tracts	22.21	15.35	29.31	22.17	63.92	30.52	24.36	14.58	32.18
DMN rsfMRI connectivity	9.10	29.84	21.08	20.66	21.45	43.90	18.91	27.59	41.65
rsMEG connectivity	10.59	6.62	11.65	15.13	32.11	17.38	22.26	25.64	25.28

BF: Bayes factor; MD: mean diffusivity; WM: white matter; DMN: default mode network; rsfMRI: resting state functional magnetic resonance imaging; rsMEG: resting state magnetoencephalography.

The column headings for each analysis are explained in the text. The value of P in the column ‘Lin = P’ was determined from the literature cited in section ‘Power calculation’, that is, P = [−0.936, −0.14, −0.56, +0.77, +1.23, +0.49] for the six phenotypic variables. The direction of the one-tailed test in the column (i.e. ‘Lin > 0’ or ‘Lin < 0’) was determined by the sign of P (i.e. negative for the first three phenotypic variables and positive for the remaining three).

In all cases, the BFs provided ‘substantial’ (BF > 3) or ‘strong’ (BF > 10) evidence (https://en.wikipedia.org/wiki/Bayes_factor) for no interaction between APOE status and age on any of the phenotypic variables, regardless of whether their prior expectation was equal to zero or equal to the effect size from the literature, with the exception of fluid intelligence: While there was strong evidence for no interaction between the ε2 allele and age on fluid intelligence, this was not true for the predicted direction of interaction between ε4 allele and age (or of overall ε4 load), where the BFs were around 2, that is, ambiguous.

## General discussion

This study provided no support for, and mostly evidence against, the ‘antagonistic pleiotropy’ hypothesis (Han and Bondi, 2008), whereby the ε4 variant of APOE is proposed to be advantageous in early life but disadvantageous in later life. The study also provided evidence against the related hypothesis that the ε2 variant of APOE is advantageous, particularly in later life (Suri et al., 2013). While our sample was relatively small for genetic analysis of cognitive and neural phenotypes, it was sufficiently powered for classical statistics when based on previous effect sizes reported, and moreover, furnished BFs that provided evidence in favour of the null hypothesis that these APOE variants do not interact (linearly) with age in their putative effects on brain structure or cognition.

The only effect of APOE status that survived our a priori correction for the six phenotypic variables tested was the main effect of ε4-carriers (more precisely, our ε4+ε2− group versus our ε3ε3 reference group) on the mean fMRI functional connectivity within the default mode network. This reflected higher connectivity for the ε4-carriers, consistent with Westlye et al. (2012). More importantly, there was no evidence that this effect interacted with age, and therefore even if it reflects a true genetic effect on brain functional connectivity, it is not evidence for the antagonistic pleiotropy hypothesis.

While all phenotypic variables showed strong associations with age, there was only one suggestion that such age associations depended on APOE status, namely, a steeper, linear age-related decline in fluid intelligence in the ε4+ε2− group than ε3ε3 group. This effect did not survive correction for multiple comparisons. Moreover, the BF for this linear effect was ambiguous, so this effect needs replication before providing support for the antagonistic pleiotropy hypothesis.

None of the analyses showed any evidence of differences for ε2-carriers (i.e. when comparing our ε4+ε2− group versus our ε3ε3 reference group). While the number of ε2-carriers was lower than ε4-carriers, the lack of interaction between ε2 and age is unlikely to simply reflect low power, because BFs provided evidence (BFs > 9) for the interaction being zero.

The classical power estimates may have been over-estimated because the effect sizes reported in the literature are likely to be inflated owing to publication bias and/or ‘winner’s curse’. Indeed, the BFs for the null hypothesis were highest when the prior mean was based on a published effect size. Nonetheless, the BF still provided substantial to strong evidence for no effect even when the prior mean was zero.

While this study had lower power than many previous studies of APOE on cognition, it is nonetheless larger than many prior APOE studies using neuroimaging measures, particularly measures of functional connectivity using fMRI or MEG, which often use small and/or biased samples of the population. Relative to these studies, this study gains power by virtue of the wide and near-uniform age range from 18 to 88 years, in a population-derived sample. Nevertheless, it is important to note that, by only considering adults, we cannot address a version of the antagonistic pleiotropy hypothesis in which the interactions between APOE status and age only occur during cognitive and brain development, that is, in individuals under 18 years. This issue could be examined in larger cognitive and neuroimaging lifespan cohorts like the European LifeBrain consortium (Walhovd et al., 2018).

Finally, there are other caveats associated with our study. First, there may be other types of bias in the selection of adults used in this study, particularly those towards the older end of our age range, for example, owing to survival bias and exclusion of those with evidence of cognitive decline. Indeed, it is possible that the effects of APOE variants are only detectable in individuals with AD pathology (e.g. Vemuri et al., 2010) and there were few such people in our cohort, even those in the pre-symptomatic phase of AD. There will also be a bias induced by the exclusion of those who were unable to undergo an MRI scan, for example, because of cardiovascular problems that require a pacemaker or stent, but which might also be related to APOE allelic status and cognitive/brain health. Second, our sample only included White Europeans (Caucasians), whereas APOE variants may have stronger effects in other genetic groups, and we did not have any direct measures of neuropathology or take into account potential effects of a range of medications. Third, this is a cross-sectional sample, which may be confounded with effects of birth-year and is unable to examine true ageing within individuals. Fourth, we cannot discount a role for antagonistic pleiotropy for phenotypic measures that we did not examine in this study such as other measures of cognition or the brain.

## Supplemental Material

APOE-CamCAN-BNA-Stage2_SupMat – Supplemental material for Effect of apolipoprotein E polymorphism on cognition and brain in the Cambridge Centre for Ageing and Neuroscience cohort Click here for additional data file. Supplemental material, APOE-CamCAN-BNA-Stage2_SupMat for Effect of apolipoprotein E polymorphism on cognition and brain in the Cambridge Centre for Ageing and Neuroscience cohort by Richard N. Henson, Sana Suri, Ethan Knights, James B. Rowe, Rogier A. Kievit, Donald M. Lyall, Dennis Chan, Else Eising and Simon E. Fisher in Brain and Neuroscience Advances
