# Supplementary material for: Effect of apolipoprotein E polymorphism on cognition and brain in the Cambridge Centre for Ageing and Neuroscience cohort
Source: Brain Neurosci Adv. 2020 Oct 7;4:2398212820961704. doi: 10.1177/2398212820961704 (PMC7545750; doi:10.1177/2398212820961704)
Supplement: APOE-CamCAN-BNA-Stage2_SupMat – Supplemental material for Effect of apolipoprotein E polymorphism on cognition and brain in the Cambridge Centre for Ageing and Neuroscience cohort [file APOE-CamCAN-BNA-Stage2_SupMat.docx]

**Supplementary Material For:**

Effect of APOE polymorphism on cognition and brain in the CamCAN cohort

Stage 2 Registered Report

Henson, R.N.^1,2,*^, Suri, S.^3,4^, Knights, E.^1^, Rowe, J.B.^1,5^, Kievit, R.A.^1^, Lyall, D.M.^6^, Chan, D.^7^, Eising, E.^8^ & Fisher, S.E. ^8,9^

^1^ MRC Cognition & Brain Sciences Unit, University of Cambridge, UK

^2^ Department of Psychiatry, University of Cambridge, UK

^3^ Department of Psychiatry, Warneford Hospital, University of Oxford, Oxford, United Kingdom

^4^ Wellcome Centre for Integrative Neuroimaging, University of Oxford, Oxford, United Kingdom

^5^ Department of Clinical Neurosciences, University of Cambridge, UK

^6^ Institute of Health and Wellbeing, University of Glasgow, Glasgow, United Kingdom

^7^ Institute of Cognitive Neuroscience, University College London, United Kingdom

^8^ Language and Genetics Department, Max Planck Institute for Psycholinguistics, Nijmegen, The Netherlands

^9^ Donders Institute for Brain, Cognition and Behaviour, Radboud University, Nijmegen, The Netherlands

* Corresponding Author:

Prof Richard Henson

MRC Cognition & Brain Sciences Unit

15 Chaucer Road

Cambridge, CB2 7EF

UK

rik.henson@mrc-cbu.cam.ac.uk

## Supplementary Table 1: Additions/minor deviations in MRI/MEG preprocessing from Stage 1 report

1. Hippocampal volume was averaged over left and right hippocampal estimates, before being corrected for TIV.
2. For the White Matter MD measure, data from 1 of the 10 tracks (Ventral Cingulate) was excluded because too many participants had missing data from this track owing to insufficient diffusion-weighted MR signal.
3. For the resting-state fMRI, the images were pre-processed as in Geerligs et al. (2017), as stated in Stage 1, and the connectivity estimated between each pair of the 18 ROIs in the dorsal and ventral default mode networks (DMNs) of Shirer et al. (2012). The connectivity estimate was the parameter fit from a linear model containing additional confounds of a discrete cosine transform (to implement a high-pass filter up to 0.008Hz), white-matter and CSF signals, and a second-order Volterra expansion of the motion parameters with lags of 0-2 TRs.
4. For the MEG, the data were processed as in Koelewijn et al. (2019), except for the following differences. Because the acquisition machine differed (having 102 magnetometers and 204 planar gradiometers rather than 275 axial gradiometers), an initial step of signal-space separation was added to remove environment noise. This also reduced the rank of the data to approximately 65, so a smaller number of ROIs were used (39), based on Colclough et al., 2015, 2016), rather than the 90 AAL ROIs used by Koelewijn et al. (2019). Also, we detected epochs with artefacts using an automated process (osl_detect_artefacts in OSL, <https://ohba-analysis.github.io/osl-docs/>), because of the much larger number of participants (and inability to know exactly what subjective criteria Koelewijn et al., 2019, used). Finally, we focused on alpha frequency (8-12Hz), since that showed the strongest effect of Age.

## Supplementary Table 2: Effects of Age on each phenotypic variable

| Poly Order | Fit | 1 | 2 |
| --- | --- | --- | --- |
| Fluid Intelligence | R^2^ =.490  df = 551 | **-2.290 (0.102)**  **T=22.5** | **-0.511**  **(0.102)**  **T=5.02** |
| Episodic Memory | R^2^ =.145  df = 584 | **-2.185 (0.213)**  **T=10.3** | -0.237  (0.213)  T=1.11 |
| Hippocampal Volume | R^2^ =.411  df = 528 | **-223.3 (12.69)**  **T=17.6** | **-105.2**  **(12.68)**  **T=8.29** |
| MD of WM tracts | R^2^ =.698  df = 474 | **1.36e-4 (4.78e-6)**  **T=28.1** | **8.66e-5**  **(4.78e-6)**  **T=18.1** |
| DMN rsfMRI connectivity | R^2^ =.046  df = 533 | **-0.013 (0.003)**  **T=5.08** | -0.003  (0.003)  T=1.05 |
| rsMEG connectivity | R^2^ =.034  df = 492 | **0.176 (0.039)**  **T=4.52** | -0.044  (0.039)  T=1.41 |

Supplementary Table 2: Results from GLM of 1^st^ (linear) and 2^nd^ (quadratic) polynomial effects of Age, from the Parametric APOE Model (i.e., all participants), for each phenotypic variable, together with the adjusted R^2^ for the whole GLM. The top number is the parameter estimate, while the number below in parentheses is the standard error of that estimate. Note that for one-tailed p<.05/6, |T|>2.40 for the minimum number of df’s here. Significant effects are shown in **bold**.

## Supplementary Table 3: Effects of APOE after adjusting for covariates

| Contrast | ε3ε3 group *vs.*  ε2+/ε4- group | | | |  | ε4+/ε2- group *vs.*  ε3ε3 group | | |  | Parametric (dose) effect of ε2-/ε4+ | | |
| --- | --- | --- | --- | --- | --- | --- | --- | --- | --- | --- | --- | --- |
| Poly. Order | Fit | 0 | 1 | 2 | Fit | 0 | 1 | 2 | Fit | 0 | 1 | 2 |
| Fluid Intelligence | **R^2^=.537**  **df=395** | 0.055  (0.113)  T=0.489 | 0.100  (0.148)  T=0.675 | -0.020  (0.148)  T=0.135 | **R^2^=.575**  **df=473** | -0.094  (0.099)  T=0.347 | -0.157  (0.107)  T=1.472 | 0.183  (0.106)  T=1.718 | **R^2^=.488**  **df=547** | -0.195  (0.102)  T=1.905 | **-0.233**  **(0.102)**  **T=2.276** | 0.157  (0.102)  T=1.536 |
| Episodic Memory | **R^2^=.217**  **df=423** | 0.071  (0.233)  T=0.303 | 0.246  (0.306)  T=0.805 | -0.091  (0.305)  T=0.297 | **R^2^=.225**  **df=502** | -0.104  (0.220)  T=0.472 | -0.141  (0.239)  T=0.472 | 0.113  (0.238)  T=0.475 | **R^2^=.151**  **df=580** | -0.154  (0.213)  T=0.723 | -0.223  (0.213)  T=1.078 | 0.030  (0.215)  T=0.139 |
| Hippocampal Volume | **R^2^=.407**  **df=382** | -24.10  (14.88)  T=1.619 | -4.706  (19.55)  T=0.241 | -17.03  (19.55)  T=0.873 | **R^2^=.414**  **df=454** | **29.22**  **(13.75)**  **T=2.124** | -4.621  (14.91)  T=0.310 | 21.201  (14.88)  T=0.081 | **R^2^=.415**  **df=524** | -7.305  (12.70)  T=0.575 | -11.18  (12.73)  T=0.878 | -9.98  (12.72)  T=0.785 |
| MD of WM tracts | **R^2^=.710**  **df=339** | 7.83e-6  (5.59-6)  T=1.401 | 4.00e-6  (7.31e-6)  T=0.547 | 8.24e-6  (7.31e-6)  T=1.123 | **R^2^=.700**  **df=407** | -1.01e-5  (5.22e-6)  T=1.94 | -5.50e-6  (5.59e-6)  T=0.984 | -4.30e-6  (5.59e-6)  T=0.769 | **R^2^=.696**  **df=470** | 3.87e-6  (4.85e-6)  T=0.798 | 4.69e-6  (4.90e-6)  T=0.959 | 1.57e-6  (4.86e-6)  T=0.324 |
| DMN rsfMRI connectivity | **R^2^=.046**  **df=383** | 1.94e-4  (2.85e-3)  T=0.007 | -2.78e-3  (3.73e-3)  T=0.744 | -2.85e-4  (3.72e-3)  T=0.765 | **R^2^=.068**  **df=458** | **7.68e-3**  **(2.77e-3)**  **T=2.770** | 3.40e-4  (2.99e-3)  T=0.114 | 3.35e-3  (2.98e-3)  T=1.126 | **R^2^=.042**  **df=529** | 4.71e-3  (2.59e-3)  T=1.820 | -1.54e-3  (2.58e-3)  T=0.595 | -2.54e-4  (2.58e-3)  T=0.912 |
| rsMEG connectivity | **R^2^=.068**  **df=352** | 0.057  (0.043)  T=1.321 | 0.038  (0.056)  T=0.676 | -0.086  (0.056)  T=1.525 | **R^2^=.065**  **df=421** | -0.034  (0.042)  T=0.817 | -0.029  (0.045)  T=0.656 | 0.009  (0.045)  T=0.200 | **R^2^=.029**  **df=488** | 0.004  (0.039)  T=0.093 | -0.007  (0.039)  T=0.192 | -0.032  (0.039)  T=0.807 |

Supplementary Table 3. GLM results for each phenotypic variable (row), after adjusting for four covariates of sex, SES, education and cardiovsascular health (see Methods for definitions). The three groups of columns refer to the planned contrasts across APOE groups. Within each group, the first column gives the overall model fit, and the next three columns give the parameter estimates, standard error (in brackets) and unsigned T-statistic for the interaction between the APOE contrast and the three polynomial expansions of age: 0^th^ (constant), 1^st^ (linear) and 2^nd^ (quadratic), where 0^th^ order term is equivalent to main effect of APOE contrast (see Supplementary Table 2 for parameters for main effects of Age). All regressors in the GLM were Z-scored. Effects with p<.05 are shown in **bold**, but note that none survived the pre-specified Bonferroni correction for 6 multiple, one-tailed comparisons (for which |T|>2.40 for the minimum number of df’s here; see text), where direction of effect was predicted to be negative for first 3 phenotypic variables and positive for last 3 phenotypic variables (see text). “Poly” = polynomial; “df” = degrees of freedom; “R^2^” = adjusted R-squared of full model.

# References

Belloy ME, Napolioni V and Greicius MD (2019) A Quarter Century of APOE and Alzheimer’s Disease: Progress to Date and the Path Forward. *Neuron* 429358. DOI: 10.1016/j.neuron.2019.01.056.

Blacker D, Haines JL, Rodes L, et al. (1997) ApoE-4 and age at onset of Alzheimer’s disease: the NIMH genetics initiative. *Neurology* 48(1): 139–47. DOI: 10.1212/wnl.48.1.139.

Buckner RL, Andrews-Hanna JR and Schacter DL (2008) The brain’s default network: Anatomy, function, and relevance to disease. *Annals of the New York Academy of Sciences*. DOI: 10.1196/annals.1440.011.

Bunce D, Anstey KJ, Cherbuin N, et al. (2012) APOE genotype and entorhinal cortex volume in non-demented community-dwelling adults in midlife and early old age. *Journal of Alzheimer’s Disease* 30(4): 935–942. DOI: 10.3233/JAD-2012-112126.

Colclough GL, Brookes MJ, Smith SM, et al. (2015) A symmetric multivariate leakage correction for MEG connectomes. *NeuroImage*. DOI: 10.1016/j.neuroimage.2015.03.071.

Colclough GL, Woolrich MW, Tewarie PK, et al. (2016) How reliable are MEG resting-state connectivity metrics? *NeuroImage*. DOI: 10.1016/j.neuroimage.2016.05.070.

Cuesta P, Garcés P, Castellanos NP, et al. (2015) Influence of the APOE ε4 Allele and Mild Cognitive Impairment Diagnosis in the Disruption of the MEG Resting State Functional Connectivity in Sources Space. *Journal of Alzheimer’s Disease* 44(2): 493–505. DOI: 10.3233/JAD-141872.

Damoiseaux JS, Seeley WW, Zhou J, et al. (2012) Gender modulates the APOE ε4 effect in healthy older adults: convergent evidence from functional brain connectivity and spinal fluid tau levels. *The Journal of neuroscience* 32(24). Society for Neuroscience: 8254–62. DOI: 10.1523/JNEUROSCI.0305-12.2012.

Davies G, Armstrong N, Bis JC, et al. (2015) Genetic contributions to variation in general cognitive function: a meta-analysis of genome-wide association studies in the CHARGE consortium (N=53 949). *Molecular Psychiatry* 20(2). Nature Publishing Group: 183–192. DOI: 10.1038/mp.2014.188.

de Mooij SMM, Henson RNA, Waldorp LJ, et al. (2018) Age Differentiation within Gray Matter, White Matter, and between Memory and White Matter in an Adult Life Span Cohort. *The Journal of Neuroscience*. DOI: 10.1523/jneurosci.1627-17.2018.

Deary IJ, Whiteman MC, Pattie A, et al. (2002) Ageing: Cognitive change and the APOE ε4 allele. *Nature* 418(6901): 932. DOI: 10.1038/418932a.

Eisenberg DTA, Kuzawa CW and Hayes MG (2010) Worldwide allele frequencies of the human apolipoprotein E gene: Climate, local adaptations, and evolutionary history. *American Journal of Physical Anthropology* 143(1). John Wiley & Sons, Ltd: 100–111. DOI: 10.1002/ajpa.21298.

Farrer LA, Cupples LA, Haines JL, et al. (1997) Effects of Age, Sex, and Ethnicity on the Association Between Apolipoprotein E Genotype and Alzheimer Disease. *JAMA* 278(16). American Medical Association: 1349. DOI: 10.1001/jama.1997.03550160069041.

Filippini N, Rao A, Wetten S, et al. (2009) Anatomically-distinct genetic associations of APOE e{open}4 allele load with regional cortical atrophy in Alzheimer’s disease. *NeuroImage* 44(3). Elsevier Inc.: 724–728. DOI: 10.1016/j.neuroimage.2008.10.003.

Filippini N, MacIntosh BJ, Hough MG, et al. (2009) Distinct patterns of brain activity in young carriers of the APOE-epsilon4 allele. *Pnas* 106(17): 7209–7214. DOI: 10.1073/pnas.0811879106.

Fleisher AS, Sherzai A, Taylor C, et al. (2009) Resting-state BOLD networks versus task-associated functional MRI for distinguishing Alzheimer’s disease risk groups. *NeuroImage* 47(4). Elsevier Inc.: 1678–1690. DOI: 10.1016/j.neuroimage.2009.06.021.

Fouquet M, Besson FL, Gonneaud J, et al. (2014) Imaging Brain Effects of APOE4 in Cognitively Normal Individuals Across the Lifespan. *Neuropsychology Review* 24(3). Springer US: 290–299. DOI: 10.1007/s11065-014-9263-8.

Fullerton SM, Clark AG, Weiss KM, et al. (2000) Apolipoprotein E variation at the sequence haplotype level: implications for the origin and maintenance of a major human polymorphism. *American journal of human genetics* 67(4): 881–900. DOI: 10.1086/303070.

Geerligs L, Rubinov M, Cam-CAN, et al. (2015) State and Trait Components of Functional Connectivity: Individual Differences Vary with Mental State. *Journal of Neuroscience* 35(41): 13949–13961. DOI: 10.1523/JNEUROSCI.1324-15.2015.

Geerligs L, Tsvetanov KA, Cam-CAN, et al. (2017) Challenges in measuring individual differences in functional connectivity using fMRI: The case of healthy aging. *Human Brain Mapping* 38(8): 4125–4156. DOI: 10.1002/hbm.23653.

Guo Y, He J, Zhao S, et al. (2014) Illumina human exome genotyping array clustering and quality control. *Nature protocols* 9(11): 2643–62. DOI: 10.1038/nprot.2014.174.

Habes M, Toledo JB, Resnick SM, et al. (2016) Relationship between APOE genotype and structural MRI measures throughout adulthood in the study of health in pomerania population-based cohort. *American Journal of Neuroradiology* 37(9). American Society of Neuroradiology: 1636–1642. DOI: 10.3174/ajnr.A4805.

Han SD and Bondi MW (2008) Revision of the apolipoprotein E compensatory mechanism recruitment hypothesis. *Alzheimer’s and Dementia* 4(4): 251–254. DOI: 10.1016/j.jalz.2008.02.006.

Heise V, Filippini N, Ebmeier KP, et al. (2011) The APOE ɛ4 allele modulates brain white matter integrity in healthy adults. *Molecular Psychiatry* 16(9). Nature Publishing Group: 908–916. DOI: 10.1038/mp.2010.90.

Ihle A, Bunce D and Kliegel M (2012) APOE ε4 and cognitive function in early life: A meta-analysis. *Neuropsychology* 26(3): 267–277. DOI: 10.1037/a0026769.

Jack CR, Wiste HJ, Weigand SD, et al. (2015) Age, sex, and APOE ϵ4 effects on memory, brain structure, and β-Amyloid across the adult life Span. *JAMA Neurology* 72(5): 511–519. DOI: 10.1001/jamaneurol.2014.4821.

Jochemsen HM, Muller M, van der Graaf Y, et al. (2012) APOE ε4 differentially influences change in memory performance depending on age. The SMART-MR study. *Neurobiology of Aging* 33(4). Elsevier Inc.: 832.e15-832.e22. DOI: 10.1016/j.neurobiolaging.2011.07.016.

Jorm AF, Mather KA, Butterworth P, et al. (2007) APOE genotype and cognitive functioning in a large age-stratified population sample. *Neuropsychology* 21(1): 1–8. DOI: 10.1037/0894-4105.21.1.1.

Koelewijn L, Lancaster TM, Linden D, et al. (2019) Oscillatory hyperactivity and hyperconnectivity in young APOE-ɛ4 carriers and hypoconnectivity in Alzheimer’s disease. *eLife* 8: 1–25. DOI: 10.7554/elife.36011.

Lancaster C, Tabet N and Rusted J (2017) The Elusive Nature of APOE ϵ4 in Mid-adulthood: Understanding the Cognitive Profile. *Journal of the International Neuropsychological Society* 23(3): 239–253. DOI: 10.1017/S1355617716000990.

Lyall DM, Royle NA, Harris SE, et al. (2013) Alzheimer’s Disease Susceptibility Genes APOE and TOMM40, and Hippocampal Volumes in the Lothian Birth Cohort 1936. Crowther DC (ed.) *PLoS ONE* 8(11). Public Library of Science: e80513. DOI: 10.1371/journal.pone.0080513.

Lyall DM, Harris SE, Bastin ME, et al. (2014) Alzheimer’s disease susceptibility genes APOE and TOMM40, and brain white matter integrity in the Lothian Birth Cohort 1936. *Neurobiology of Aging* 35(6). Elsevier Inc.: 1513.e25-1513.e33. DOI: 10.1016/j.neurobiolaging.2014.01.006.

Lyall DM, Ward J, Ritchie SJ, et al. (2016) Alzheimer disease genetic risk factor APOE e4 and cognitive abilities in 111,739 UK Biobank participants. *Age and Ageing* 45(4): 511–517. DOI: 10.1093/ageing/afw068.

Lyall Donald M., Celis-Morales C, Lyall LM, et al. (2019) Assessing for interaction between APOE ε4, sex, and lifestyle on cognitive abilities. *Neurology* 92(23). NLM (Medline): e2691–e2698. DOI: 10.1212/WNL.0000000000007551.

Lyall Donald M, Cox SR, Lyall LM, et al. (2019) Association between APOE e4 and white matter hyperintensity volume, but not total brain volume or white matter integrity. *Brain Imaging Behav.* Brain Imag. DOI: 10.1007/s11682-019-00069-9.

Machulda MM, Jones DT, Vemuri P, et al. (2011) Effect of APOE ε4 status on intrinsic network connectivity in cognitively normal elderly subjects. *Archives of Neurology* 68(9): 1131–1136. DOI: 10.1001/archneurol.2011.108.

Marioni RE, Campbell A, Scotland G, et al. (2016) Differential effects of the APOE e4 allele on different domains of cognitive ability across the life-course. *European Journal of Human Genetics* 24(6). Nature Publishing Group: 919–923. DOI: 10.1038/ejhg.2015.210.

Mondadori CRA, De Quervain DJF, Buchmann A, et al. (2007) Better memory and neural efficiency in young apolipoprotein E ε4 carriers. *Cerebral Cortex* 17(8): 1934–1947. DOI: 10.1093/cercor/bhl103.

O’Donoghue MC, Murphy SE, Zamboni G, et al. (2018) APOE genotype and cognition in healthy individuals at risk of Alzheimer’s disease: A review. *Cortex* 104. Elsevier Ltd: 103–123. DOI: 10.1016/j.cortex.2018.03.025.

Oberlin LE, Manuck SB, Gianaros PJ, et al. (2015) Blood pressure interacts with APOE ε4 to predict memory performance in a midlife sample. *Neuropsychology* 29(5): 693–702. DOI: 10.1037/neu0000177.

Pietzuch M, King AE, Ward DD, et al. (2019) The Influence of Genetic Factors and Cognitive Reserve on Structural and Functional Resting-State Brain Networks in Aging and Alzheimer’s Disease. *Frontiers in aging neuroscience* 11. Frontiers Media SA: 30. DOI: 10.3389/fnagi.2019.00030.

Radmanesh F, Devan WJ, Anderson CD, et al. (2014) Accuracy of imputation to infer unobserved APOE epsilon alleles in genome-wide genotyping data. *European Journal of Human Genetics* 22(10). Nature Publishing Group: 1239–1242. DOI: 10.1038/ejhg.2013.308.

Salgado JF (2018) Transforming the Area under the Normal Curve (AUC) into Cohen’s d, Pearson’s r pb , Odds-Ratio, and Natural Log Odds-Ratio: Two Conversion Tables. (June). DOI: 10.5093/ejpalc2018a5.

Schiepers OJG, Harris SE, Gow AJ, et al. (2012) APOE E4 status predicts age-related cognitive decline in the ninth decade: longitudinal follow-up of the Lothian Birth Cohort 1921. *Molecular psychiatry* 17(3): 315–24. DOI: 10.1038/mp.2010.137.

Schultz MR, Lyons MJ, Franz CE, et al. (2008) Apolipoprotein E genotype and memory in the sixth decade of life. *Neurology* 70(19 PART 2): 1771–1777. DOI: 10.1212/01.wnl.0000286941.74372.cc.

Shafto M a, Tyler LK, Dixon M, et al. (2014) The Cambridge Centre for Ageing and Neuroscience (Cam-CAN) study protocol: a cross-sectional, lifespan, multidisciplinary examination of healthy cognitive ageing. *BMC neurology* 14(1): 204. DOI: 10.1186/s12883-014-0204-1.

Sheline YI, Morris JC, Snyder AZ, et al. (2010) APOE4 allele disrupts resting state fMRI connectivity in the absence of amyloid plaques or decreased CSF Aβ42. *The Journal of neuroscience : the official journal of the Society for Neuroscience* 30(50). Society for Neuroscience: 17035–40. DOI: 10.1523/JNEUROSCI.3987-10.2010.

Shin MH, Kweon SS, Choi JS, et al. (2014) The effect of an APOE polymorphism on cognitive function depends on age. *Journal of Neurology* 261(1): 66–72. DOI: 10.1007/s00415-013-7157-y.

Shirer WR, Ryali S, Rykhlevskaia E, et al. (2012) Decoding subject-driven cognitive states with whole-brain connectivity patterns. *Cerebral Cortex*. DOI: 10.1093/cercor/bhr099.

Shu H, Shi Y, Chen Gang, et al. (2016) Opposite Neural Trajectories of Apolipoprotein E ϵ4 and ϵ2 Alleles with Aging Associated with Different Risks of Alzheimer’s Disease. *Cerebral cortex (New York, N.Y. : 1991)* 26(4): 1421–1429. DOI: 10.1093/cercor/bhu237.

Siebner HR, Callicott JH, Sommer T, et al. (2009) From the genome to the phenome and back: Linking genes with human brain function and structure using genetically informed neuroimaging. *Neuroscience*, 24 November. DOI: 10.1016/j.neuroscience.2009.09.009.

Stebbins GT and Murphy CM (2009) Diffusion Tensor Imaging in Alzheimer’s Disease and Mild Cognitive Impairment. *Behavioural Neurology* 21(1–2). Hindawi Limited: 39. DOI: 10.3233/BEN-2009-0234.

Suri S, Heise V, Trachtenberg AJ, et al. (2013) The forgotten APOE allele: A review of the evidence and suggested mechanisms for the protective effect of APOE e2. *Neuroscience and Biobehavioral Reviews* 37(10). Elsevier Ltd: 2878–2886. DOI: 10.1016/j.neubiorev.2013.10.010.

Taylor JL, Scanlon BK, Farrell M, et al. (2015) APOE-epsilon4 and aging of medial temporal lobe gray matter in healthy adults older than 50 years. *Neurobiol Aging* 35(11). DOI: 10.1038/jid.2014.371.

Taylor JR, Williams N, Cusack R, et al. (2015) The Cambridge Centre for Ageing and Neuroscience (Cam-CAN) data repository: Structural and functional MRI, MEG, and cognitive data from a cross-sectional adult lifespan sample. *NeuroImage*. Elsevier B.V. DOI: 10.1016/j.neuroimage.2015.09.018.

Trachtenberg AJ, Filippini N, Ebmeier KP, et al. (2012) The effects of APOE on the functional architecture of the resting brain. *NeuroImage* 59(1). Elsevier Inc.: 565–572. DOI: 10.1016/j.neuroimage.2011.07.059.

Tsvetanov K a., Henson RN a., Tyler LK, et al. (2016) Extrinsic and Intrinsic Brain Network Connectivity Maintains Cognition across the Lifespan Despite Accelerated Decay of Regional Brain Activation. *Journal of Neuroscience* 36(11): 3115–3126. DOI: 10.1523/JNEUROSCI.2733-15.2016.

Tsvetanov KA, Henson RNA, Tyler LK, et al. (2015) The effect of ageing on fMRI: Correction for the confounding effects of vascular reactivity evaluated by joint fMRI and MEG in 335 adults. *Human Brain Mapping* 36(6). DOI: 10.1002/hbm.22768.

Vemuri P, Wiste HJ, Weigand SD, et al. (2010) Effect of Apolipoprotein E on Biomarkers of Amyloid Load and Neuronal Pathology in Alzheimer Disease. 67(3): 308–316. DOI: 10.1002/ana.21953.Effect.

Walhovd KB, Fjell AM, Westerhausen R, et al. (2018) Healthy minds 0–100 years: Optimising the use of European brain imaging cohorts (“Lifebrain”). *European Psychiatry*. DOI: 10.1016/j.eurpsy.2017.12.006.

Westlye ET, Lundervold A, Rootwelt H, et al. (2011) Increased hippocampal default mode synchronization during rest in middle-aged and elderly APOE ε4 carriers: relationships with memory performance. *The Journal of neuroscience : the official journal of the Society for Neuroscience* 31(21). Society for Neuroscience: 7775–83. DOI: 10.1523/JNEUROSCI.1230-11.2011.

Westlye LT, Reinvang I, Rootwelt H, et al. (2012) Effects of APOE on brain white matter microstructure in healthy adults. *Neurology* 79(19). Wolters Kluwer Health, Inc. on behalf of the American Academy of Neurology: 1961–9. DOI: 10.1212/WNL.0b013e3182735c9c.

Wisdom NM, Callahan JL and Hawkins KA (2011) The effects of apolipoprotein E on non-impaired cognitive functioning: A meta-analysis. *Neurobiology of Aging* 32(1). Elsevier: 63–74. DOI: 10.1016/J.NEUROBIOLAGING.2009.02.003.
